# Kinin-B1 Receptor Stimulation Promotes Invasion and is Involved in Cell-Cell Interaction of Co-Cultured Glioblastoma and Mesenchymal Stem Cells

**DOI:** 10.1038/s41598-018-19359-1

**Published:** 2018-01-22

**Authors:** Mona N. Oliveira, Micheli M. Pillat, Helena Motaln, Henning Ulrich, Tamara T. Lah

**Affiliations:** 1 Department of Biochemistry, Institute of Chemistry, University of São Paulo, Av. Prof. Lineus Prestes 748, São Paulo, SP 05508-000 Brazil; 2 Department of Genetic Toxicology and Cancer Biology, National Institute of Biology, Večna pot 111, 1000 Ljubljana, Slovenia; 3 Jožef Stefan International Postgraduate School, Jamova 39, 1000 Ljubljana, Slovenia; 4 Department of Biochemistry, Faculty of Chemistry and Chemical Engineering, University of Ljubljana, Večna pot 113, 1000 Ljubljana, Slovenia

## Abstract

Glioblastoma multiforme (GBM) represents the most lethal brain tumour, and these tumours have very limited treatment options. Mesenchymal stem cells (MSC) are considered as candidates for advanced cell therapies, due to their tropism towards GBM, possibly affecting their malignancy, thus also representing a potential therapeutic vector. Therefore, we aimed to compare the effects of bone-marrow-derived *versus* adipose-tissue-derived MSC (BM-/AT-MSC) on heterogeneous populations of tumour cells. This cells’ interplay was addressed by the *in-vitro* two-dimensional (monolayer) and three-dimensional (spheroid) co-culture models, using U87 and U373 GBM cell lines, expressing genotypically different mesenchymal transcriptome profiles. U87 cell low mesenchymal profile expressed high levels of kinin receptor 1 (B1R) and their invasion was greatly enhanced by the B1R agonist des-Arg^9^-bradykinin upon BM-MSC co-culturing in 3D co-cultures. This correlated to significantly higher cell-cell interactions in U87/BM-MSC mixed spheroids. This was not observed with the U373 cells and not in AT-MSC co-cultures. Altogether, these data support the on-going exploration of B1R as target for adjuvant approach in GBM therapy. Secondly, the results emphasize the need for further careful exploration of the selectivity regarding the origin of MSC as potential candidates for cell therapies, particular in cancer, where they may adversely affect heterogeneous tumour cell populations.

## Introduction

Over the past few years, it has become evident that the tumour microenvironment is important for regulation of tumour progression^1^. Glioblastoma (GBM) is the most frequent and lethal neoplasia among brain tumours, and a vast body of literature refers to these malignant tumour cells^2^. In contrast, the dynamics and interactions of GBM cells with stromal cells within the tumour microenvironment need still to be explored. Among infiltrating glial cells/macrophages and other immune cells, astrocytes and endothelial cells, it has been shown that mesenchymal stem cells (MSC) also actively move to and reside in GBM tumours^3,4^. Tumour-infiltrating MSC have been associated with enhancement of malignancy and with induction of GBM cell proliferation and migration^5,6^. The anti-tumour effects of MSC are well known, and these include inhibition of proliferation and promotion of apoptosis and senescence of cancer cells (reviewed in^7,8^).

We have previously demonstrated that *in-vitro* cross-talk between bone-marrow-derived MSC (BM-MSC) and U87 GBM cells in an ‘indirect’ co-culture model (i.e., cells sharing medium, without direct cell-cell contact) resulted in their altered expression of over 500 and 300 genes, respectively^9^. On the other hand, in direct co-cultures of MSC and U373 GBM cells (i.e., in direct cell-cell contact), an enhancement of migration of both cell types was reported by Schichor *et al*.^3^. These authors also demonstrated that U373 cells can form structural and functional syncytium with MSC, which enables direct molecular exchange, and might also be accelerated by cell heterotypic fusion^10^ to form mixed hybrids. Recently, we addressed the effects of co-culturing MSC with two different GBM cell subtypes, as U87 and U373 cells, and we showed that the effects of MSC on glioma cells are subtype dependent^11^.

Bradykinin (BK) has been shown to regulate proliferation and to promote migration of neural stem cells and neurogenic differentiation^12,13^. However, the role of kinins in the malignant brain microenvironment remains poorly explored. Kinins are generated by kininogen processing during inflammation, and they have diverse functions, which include regulation of neuroinflammation^14^, endothelial cell activation and vasodilatation^15^. This kinin signalling system is associated with activation of proteases, which produce pharmacologically active BK and kallidin, as well as des-Arg^9^-bradykinin (DBK) and des-Arg^9^-kallidin, having pro-inflammatory effects upon binding to the two kinin receptors, B1R and B2R, respectively^16^. The B2R is constitutively expressed and has high affinity for the binding of BK and kallidin peptides; in contrast, B1R is an inducible receptor with affinity for DBK and des-Arg^9^-kallidin. In cancer, these kinin receptors are involved in cell proliferation, chemotaxis and invasion^17^, such as in breast carcinoma^18^ and glioma cells^19^. Lu and co-workers^19^ revealed that B1R expression up-regulation in GBM cells increases the cell proliferation mediated by DBK and BK. These might be induced by inflammatory cytokines secreted by tumour-infiltrating immune cells, with signalling via the phosphatidylinositol-4,5-bisphosphate 3-kinase (PI3K)/Akt (protein kinase B) and extracellular signal-regulated kinase (ERK) pathways. As MSC also have immunomodulatory properties^9,20^, immunomodulation by MSC might also induce B1R expression.

Pillat *et al*.^21^ previously showed that both B1R and B2R are expressed at the mRNA and protein levels in U87 cells and in BM-MSC *in vitro*. Additionally, they demonstrated that in indirect and direct co-cultures of U87 cells with BM-MSC, B1R gene and protein expression increased in U87 cells, which was correlated with their enhanced invasiveness via induction of metalloproteases. Here, we aimed to examine B1R involvement in direct cross-talk between two distinct GBM phenotypes (U87 and U373 cells) and MSC of two different origins (BM-MSC and adipose-tissue-derived, AT-MSC). We demonstrate differential responses of U87 and U373 GBM cells to MSC in terms of cell proliferation, cell cycle and GBM/MSC cell-cell interaction (vesicle transfer, fusion events and entosis), B1R agonist des-Arg^9^-BK stimulation of U87/BM-MSC mixed spheroids and increased migration/invasion of U87 cells. To the best of our knowledge, this is the first report on B1R as a potential regulator of these processes. Possible clinical applications of our findings are supported by studies that have already suggested B1R as biomarker^22^ for targeting in cancer therapy^17,23^. In addition, the present study has important implications for potential MSC therapies for GBM, providing mechanisms for their their interactions with tumour cells^24^.

## Results

### Cell proliferation, cell size and the cell cycle effects in mixed spheroids of glioblastoma and bone-marrow-derived mesenchymal stem cells

**Figure 1:**
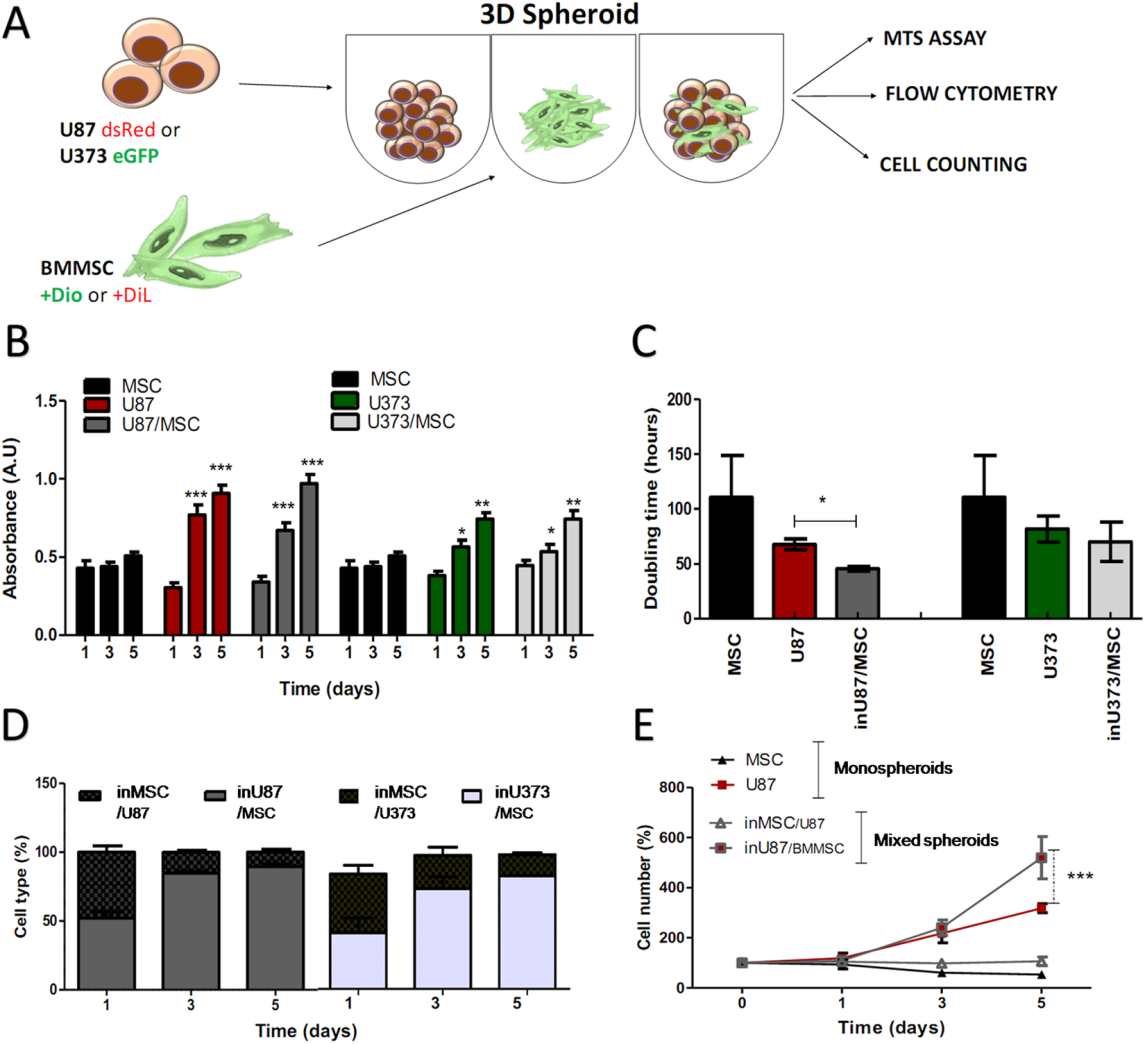
Bone-marrow (BM) MSC induce proliferation of GBM cells in mono-cultures and mixed spheroids. (**A**) Experimental scheme for mono-spheroids and mixed (co-cultured) spheroids for the studies of phenotype changes. (**B**) Total cell viability presented as metabolic activity (A.U. at λ 490/630 nm) in mono cultures of U87dsRed and U373eGFP GBM cells, and BM-MSC (=MSC), and in mixed spheroids (U87/MSC, U373/MSC), as determined by the MTS assay after 1, 3, and 5 days (see Methods). (**C**) Mean doubling times (h) as calculated from the cell counts (see Methods), for U87 and U373 cells, and MSC, in mono-spheroids and mixed spheroids. (**D**) Percentages of GBM cells and MSC in mixed spheroids, marked as in U87/MSC and inU373/MSC were determined by flow cytometry after 1, 3, and 5 days in co-culture. (**E**) Proliferation of U87 cells and MSC in mono-spheroids and mixed spheroids, as calculated from the cell counting after 1, 3, and 5 days, and normalized to the number of cells present in the spheroids on day 0. Data are means ± standard deviation (n = 3); *P < 0.05, **P < 0.001, ***P < 0.0001.

The work-flow for the (co-)culturing of the cells as three-dimensional (3D) mono-cultures (termed ‘mono-spheroids’ of a single cell type) and mixed 3D co-cultures (termed ‘mixed spheroids’, formed from different cell types mixed in a 1:1 ratio) is shown in Fig. 1A. Briefly, these spheroids were formed as mono or mixed cells seeded with 4% methylcellulose in 96-well U-bottomed plates (2.5 × 10^3^cells/well; see Methods for details). These were left in culture for 1 day at 37 °C under 5% CO_2_, to form one spheroid per well.

The proliferation rates of the U87dsRed and U373eGFP GBM cells and BM-MSC as mono-spheroids and mixed spheroids were assessed for their viability using the 3-(4,5-dimethylthiazol-2-yl)-5(3-carboxymethonyphenol)-2-(4-sulfophenyl)-2H-tetrazolium (MTS) as say in terms of the metabolic activity of the cells (Fig. 1B), and for their population doubling times in terms of automated cell counting (Fig. 1C). These U87dsRed and U373eGFP GBM cells showed increased proliferation rates as mixed spheroids with BM-MSC, as compared to their mono-spheroid cultures. As BM-MSC proliferation was slower compared to these GBM cells, as shown by the lower metabolic activity of the BM-MSC *versus* GBM mono-spheroids, we can conclude that in our experimental condition the metabolically more active and proliferating GBM cells accounted for the increased metabolic activity of these GBM/BM-MSC mixed spheroids (Fig. 1B). Furthermore, the U87dsRed cells were more proliferative as both mono-spheroids and mixed spheroids than the U373eGFP cells (Fig. 1B). BM-MSC-induced proliferation of U87dsRed cells significantly halved their population-doubling time, showing a greater effect than that seen for U373eGFP cells (Fig. 1C). The predominance of U87dsRed cells in these mixed spheroids was confirmed using flow cytometry over this period of 5 days (Fig. 1D), and similar, although smaller, increases in U373eGFP cells over BM-MSC in the mixed spheroids were also seen. Direct cell counts confirmed that the U87dsRed cell numbers increased in the U87/BM-MSC mixed spheroids with time (up to 5 days), whereas the number of BM-MSC cell in these mixed spheroids decreased (Fig. 1E).

**Figure 2:**
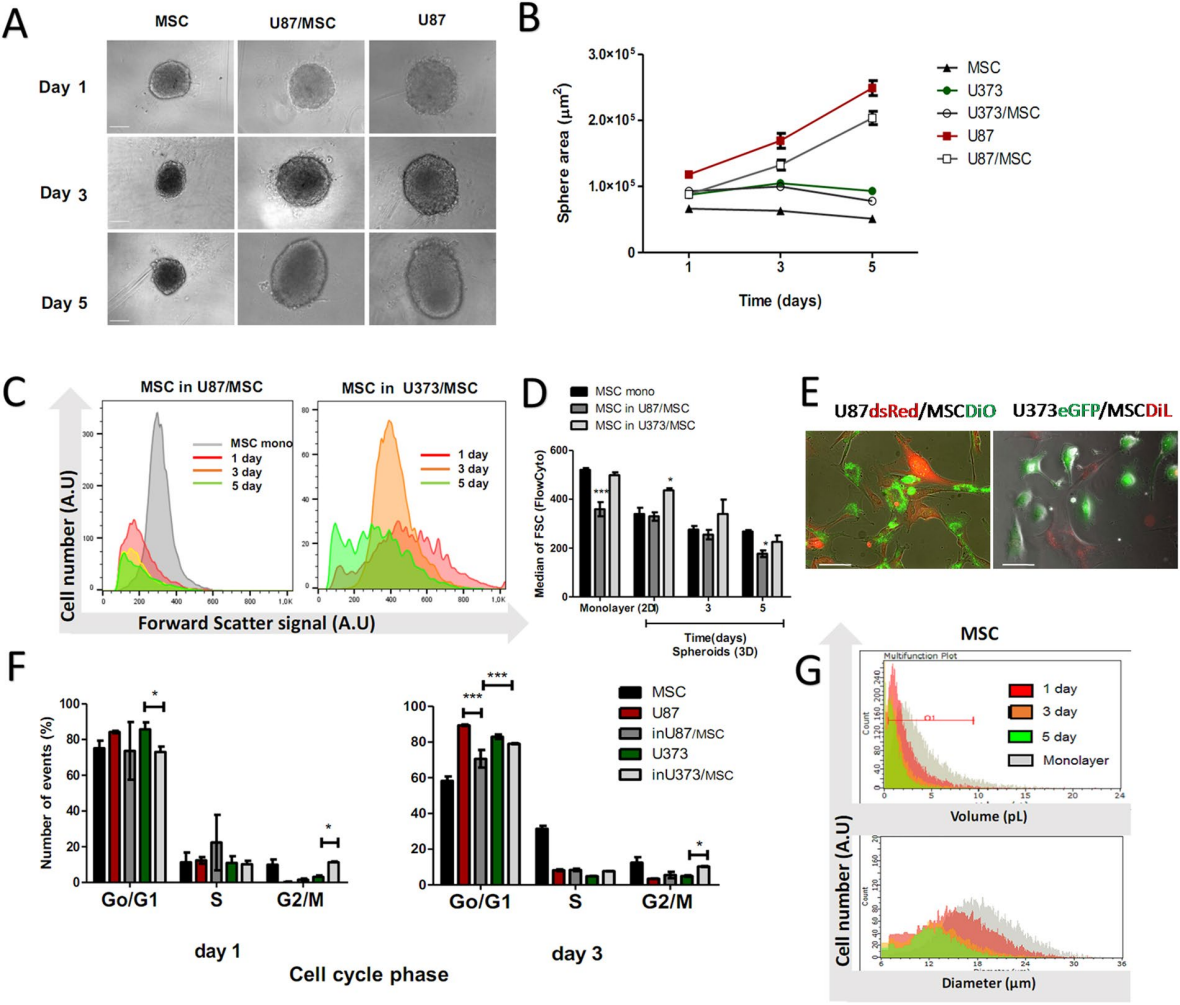
Bone-marrow MSC change in size and morphology when co-cultured with GBM cells of different origins. (**A**) Representative images of spheroids’ sizes of BM-MSC (=MSC), U87/MSC, and U87 spheroids. (**B**) Quantification of the spheroids area size after 1, 3, and 5 days in culture, performed on micrographs using the NIS elements software, as described in Methods. (**C**) Changes in MSC area size in co-cultures with U87 and U373 cells after 1, 3, and 5 days in culture (increased forward scatter meaning increased cell size, as described in Methods. (**D**) Changes in cell size of MSC were quantified in 3D mono-spheroids and in mixed spheroids with U87 and U373 cells, after 1, 3, and 5 days in culture. The control MSC cell sizes were measured in 2D monolayer cultures and 2D co-cultures is shown (the first bar graph, left). (**E**) Representative phase-contrast microscopy images of cell morphology in 2D monolayer cultures of U87dsRed (in red) and U373eGFP (in green) GBM cells with MSC DiO (in green) and MSC DiL (in red) respectively; scale bar, 25 µm. (**F**) Quantification for cell cycle analyses of U87 and U373 cells and MSC as 2D monolayer cultures and co-cultures, for 1 and 3 days. (**G**) Representative data for diameter and volume measurements of MSC grown as 2D monolayer and as spheroid after 1, 3, and 5 days, determined by Scepter cell counting device. Data are means ± standard deviation (n = 3). *P < 0.05, ***P < 0.0001.

Figure 2 shows comparisons of the spheroid sizes after 3 and 5 days of co-culturing of the GBM cells with BM-MSC, using imaging and flow cytometry analysis of mono-spheroid and mixed spheroid cultures (Fig. 2A,B). U87dsRed mono-spheroids and U87/BM-MSC mixed spheroids increased their cross-sectional areas up to 5 days, whereas those for BM-MSC mono-spheroids and U373 and U373/BM-MSC mixed spheroids decreased (Fig. 2B). This decrease in the BM-MSC spheroid size paralleled the BM-MSC cell size decrease that was determined through the forward scattering of the BM-MSC as both mono-spheroids and mixed spheroids (Fig. 2C,D). The BM-MSC were becoming significantly smaller in size when cultured with the U87dsRed GBM cells in both 2D (monolayer) and in these 3D (spheroid) cultures in a time dependent manner (Fig. 2C–E,G). On the other hand, the BM-MSC in the U373/BM-MSC mixed spheroids did not decrease in size. Also, no changes were detected for the U87dsRed and U373eGFP GBM cell sizes when cultured as mono-spheroids and mixed spheroids. The GBM and BM-MSC cell cycle alterations were also followed after 2D (monolayer) co-culturing. These analyses confirmed that after 3 days, for U87dsRed cells in co-culture with BM-MSC there was a small but significant decrease in the G0/G1 phase population (Fig. 2F, right panel), where as the U373eGFP cells in co-culture with BM-MSC showed increased cell population in the G2/M phase already from the first day (Fig. 2F).

Taken together, this supports the data showing that BM-MSC increase the proliferation of U87dsRed cells in mixed co-cultures more than seen for U373eGFP cells in mixed co-cultures. However, there were no changes in the GBM cell sizes, in contrast to BM-MSC, that were becoming particularly smaller in co-cultures with U87dsRed cells, but not with U373eGFP cells.

### Glioblastoma and mesenchymal stem cell-cell interaction rate is cell-type dependent

Tumour cells have been reported to fuse with each other (i.e., to form homotypic hybrids) and to fuse with stromal cells (i.e. to form heterotypic hybrids)^10,25,26^. Vesicle-mediated transfer also are fundamental biological processes that are emerging as novel mechanisms for re-programming cells in the tumour microenvironment^27^. The question here was whether the cell-cell interaction (vesicle transfer and cell fusion) might be cell-phenotype dependent. Thus, the potential of these phenotypically different GBM cell lines, U87 and U373, to form heterotypic hybrids and vesicle transfer with BM-MSC was studied.

**Figure 3:**
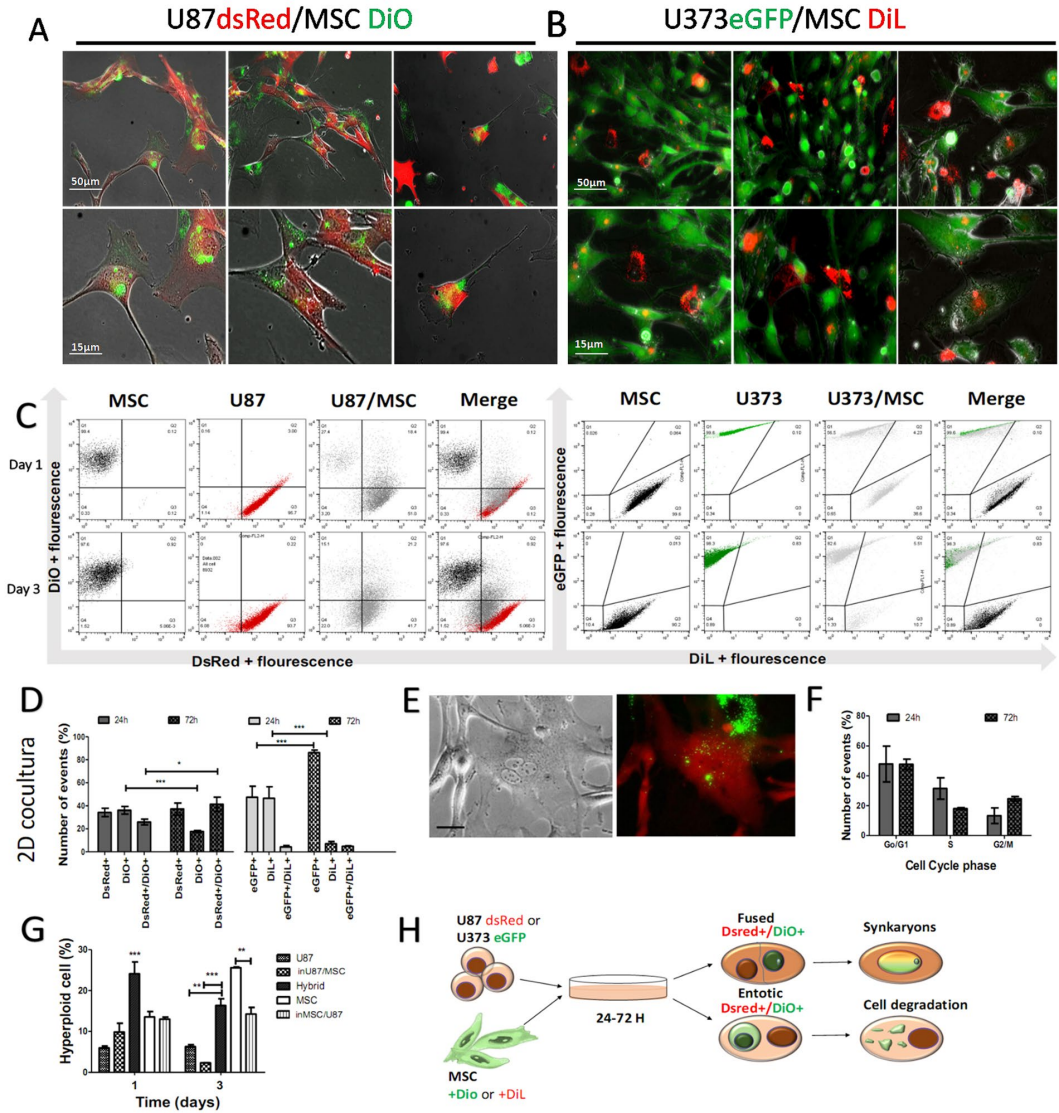
Cell-Cell interaction events in 2D monolayer co-cultures of GBM U87 and U373 cells with BM-MSC. (**A**) Representative images of heterotypic cell fusion (orange) between U87-dsRed cells (red) and BM-MSC (=MSC; green), indicated by arrow heads after 3 days in co-culture. (**B**) As for (**A**), between U373-GFP cells (green) and MSC; red) and rare events of cell fusion (orange). (**C**) Representative flow cytometry dot-plots after 1 and 3 days of co-culturing, showing the increase in the cell-cell interaction populations in the last (right) panel (Merge): Ds Red+= U87 in U87/MSC, DiO+= MSC in U87/MSC Dared+/DiO+= hybrid cells, entosis or vesicle transfer; eGFP+= U373 in U373/MSC, Dil+= MSC in U373/MSC eGFP+/Dil+= hybrid cells, entosis or vesicle transfer. (**D**) Quantification of cell-cell interaction events (cell hybrids) in U87/MSC and U373/MSC mixed spheroids, as derived from flow cytometry data in (**C**). (**E**) Representative images of heterotypic fusion or entotic events between U87dsRed cells (red) and MSCs (green) as 2D monolayer co-cultures, after 3 days. Scale bar, 20 µm. (**F**) Cell cycle analysis of the fused U87/BM-MSC cells in 2D monolayer co-cultures after 1 (24 h) and 3 (72 h) days. (**G**) Polyploidy of cell populations (quantified as described in Methods) in U87/MSC 2D monolayer co-cultures, after 1 and 3 days. (**H**) Scheme of the fusion or entotic events between the different types of cells, under the present experimental set-up. For quantifications, data are means ± standard deviations (n = 3). *P < 0.05, **P < 0.001, ***P < 0.0001.

U87 and U373 cells expressing red fluorescence protein (dsRed) and green fluorescent protein (GFP), respectively, and BM-MSC pre-labelled with the vital fluorescent dyes DiO and DiI were grown as 2D monolayers as single cell types (2D mono-cultures) and mixed cell types (2D co-cultures) for 3 days, and then cell-cell interactions were imaged by fluorescence microscopy and flow cytometry, as described in the Methods. Figure 3(A,B) shows merged images of U87-dsRed cell-cell interaction with BM-MSC-DiO cells, recorded as the double fluorescent signals. Some of these hybrid cells retained double nuclei (i.e., green and red) derived from both cell types, and had either mosaic membrane (red and green). The frequency of cell-cell interaction of these cells in the 2D co-cultures apparently depended on the GBM cell phenotype: for U87-dsRed/BM-MSC-DiO 2D co-cultures after 1 day, cell-cell interaction were more abundant (25.8 ± 6.6%), when compared to U373-GFP/BM-MSC-DiL2D co-cultures (4.2 ± 2.0%). Moreover, the percentage of those events increased after 3 days in U87dsRed/BM-MSC-DiO2D co-cultures (41.4 ± 15.1%), whereas cell-cell interaction remained low in U373-GFP/BM-MSCDiL2D co-cultures (4.9 ± 0.8%) (Fig. 3C,D). This thus suggests that compared to U373 cells, U87 cells (which have been shown to have a less pronounced mesenchymal phenotype) have greater tendency to cell-cell interaction with vesicle transfer or fuse with the BM-MSC to form heterotypic hybrids and eventual entosis events (Supplementary Fig. 1). Tumour cells showing multiple nuclei and double labelling for dsRed and DiO after 3 days in these 2D co-culture are shown in Fig. 3E. Cell-cycle analyses performed after 1 and 3 days revealed that the U87-dsRed/BM-MSC-DiO double staining population in S phase decreased, whereas the proportion of these cell in G2/M phase increased after 3 days (Fig. 3F). The G2/M phase increase is most likely be assigned to mitosis ploidy changes, as high levels of polyploidy persisted in the double labeled populations at both 1 and 3 days (Fig. 3G). Contrary to high polyploidy found in these double-labeled populations, polyploidy in 2D co-cultured U87 cells and BM-MSC decreased after 3 days, compared to U87 cells and BM-MSC grown alone in 2D mono-cultures. These results suggest that through cell-cell interaction heterotypic fusion occurs, possibly involving transition from heterokaryon to syncharyon. Entosis, the process of the invasion of one cell into another might also have occurred here (Fig. 3E,H), although we have not distinguished both processes by molecular markers, as suggested by Sottile *et al*.^28^ Altogether, these data suggest that U87 and U373 have distinct cell-cell interaction potentials, which are possibly linked to their differentially expressed mesenchymal phenotypes.

### Glioblastoma U87 dsRed cells acquire mesenchymal phenotype when co-cultured with BM-MSC

**Figure 4:**
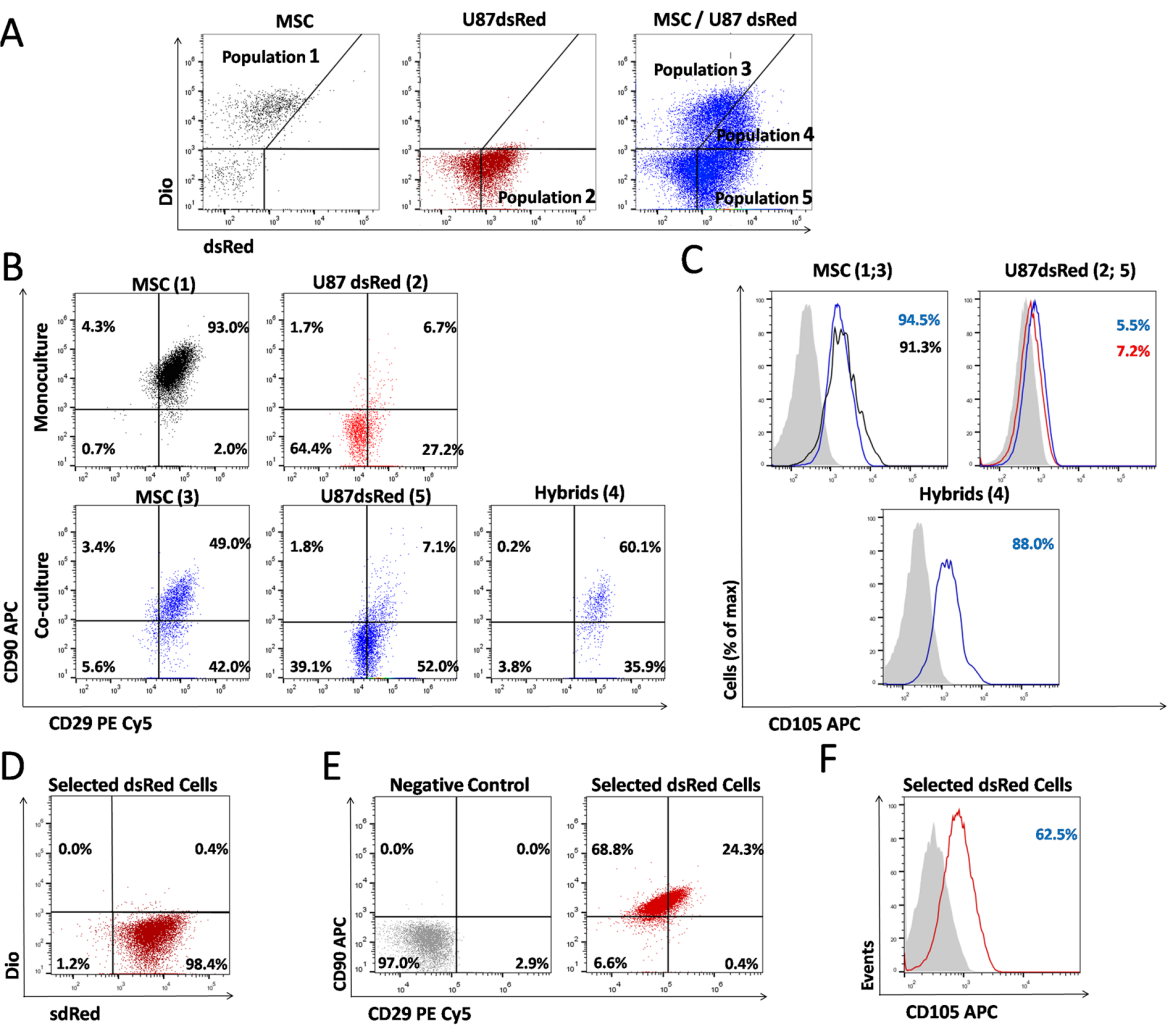
Glioblastoma U87dsRed cells acquire the mesenchymal phenotype upon co-culturing with MSC. (**A**) Flow cytometry dot-plots after 3 days of mono- and co-culture, DiO^+^ MSC (population 1 in monoculture and population 3 in co-culture), dsRed^+^ U87 cells (population 2 in monoculture and population 5 in co-culture), and DiO^+^ dsRed^+^ hybrid cells (population 4 in co-culture). **(B**) Dot-plots for mesenchymal stem cell markers CD90 and CD29 in MSC and U87 cells in mono- and co-culture, demonstrating that the hybrid population 4 is positive for CD90 and CD29, such as observed in MSC (populations 1 and 3). (**C**) Flow cytometry histograms, comparing expression of CD105 in MSC (upper left, monoculture in black line and co-culture in blue line); CD105 expression in U87 cells (upper right (monoculture in red line and co-culture in blue line), CD105 positivity in hybrid cells (lower histogram; co-culture in blue line. The data show expression of the MSC related marker also in hybrid cells. Gray histograms represent negative controls, as described in Methods. (**D**) Selection of U87 dsRed cell by geneticin treatment for 6 days, in co-cultures with MSC was efficiently selecting for 98.4% of dsRed^+^DiO^−^ cells. (**E**) Flow cytometry dot-plots of these selected dsRed cells evidenced high expression rates of CD90, CD29 and (**F**) CD105. Data were analyzed with Flowjo V10 software.

We investigated the expression of mesenchymal-specific membrane markers, CD29, CD90 and CD105 in monoculture and co-culture of BM-MSC and U87dsRed cells by flow cytometry (Fig. 4). As expected, the majority (90%) of BM-MSC were highly positive for these markers, of which CD90 expression in BM-MSC decreased upon co-cultures from 93% to 49% (Fig. 4B), but a reduction of CD29 (97.3 to 91.0%) and CD105 (94.5 to 91.3%) was not significant (Fig. 4B,D). As expected, U87 cells expressed very low levels of these markers, not being significantly altered upon co-culturing for CD90 (from 8.4% to 8.9%) and CD105 (from 5.5% to 7.2%), but was significantly increased for CD29 from 33.9% to 59.1%.

The double stained dsRed^+^/DiO^+^ cells in co-cultures of U87dsRed/BM-MSCDiO, were separated after 3 days as population 4 in Fig. 4A, and the frequencies of CD29, CD90 and CD105 were again quantified by flow cytometry analysis, revealing that the double stained cells expressed higher levels of mesenchymal cell-specific markers, such as 88% of CD105, 96% of CD29, and 60% of CD90 (Fig. 4B,E). These data demonstrated that U87dsred/BM-MSCDiO double stained population expressed higher mesenchymal phenotype. This may enhance U87 migration, possibly triggered by cytokines secreted from mesenchymal stem cells^20,29,30^. Furthermore, after 6 days in co-culture, U87dsRed cells were selected by geneticin (G418), 1 mg/ml treatment to observe the CD29, CD90 and CD105 levels within the 2D coculture. As shown in Fig. 4D, G418 selected for survival of 98.4% U87dsRed cells, but no MSCDiO was present. Interestingly, some of these selected U87dsRed cells, presumably containing both U87 transdifferentiated and hybrid cell population, expressed rather high levels of the mesenchymal-specific markers, 24.7% of CD29, 93.1% of CD90, and 62.5% of CD105 (Fig. 4E,F).

### B1R expression and alterations in U87 and U373 glioblastoma cells in response to mesenchymal stem cells are associated with heterotypic cell fusion and vary in MSC of different tissue origin

**Figure 5:**
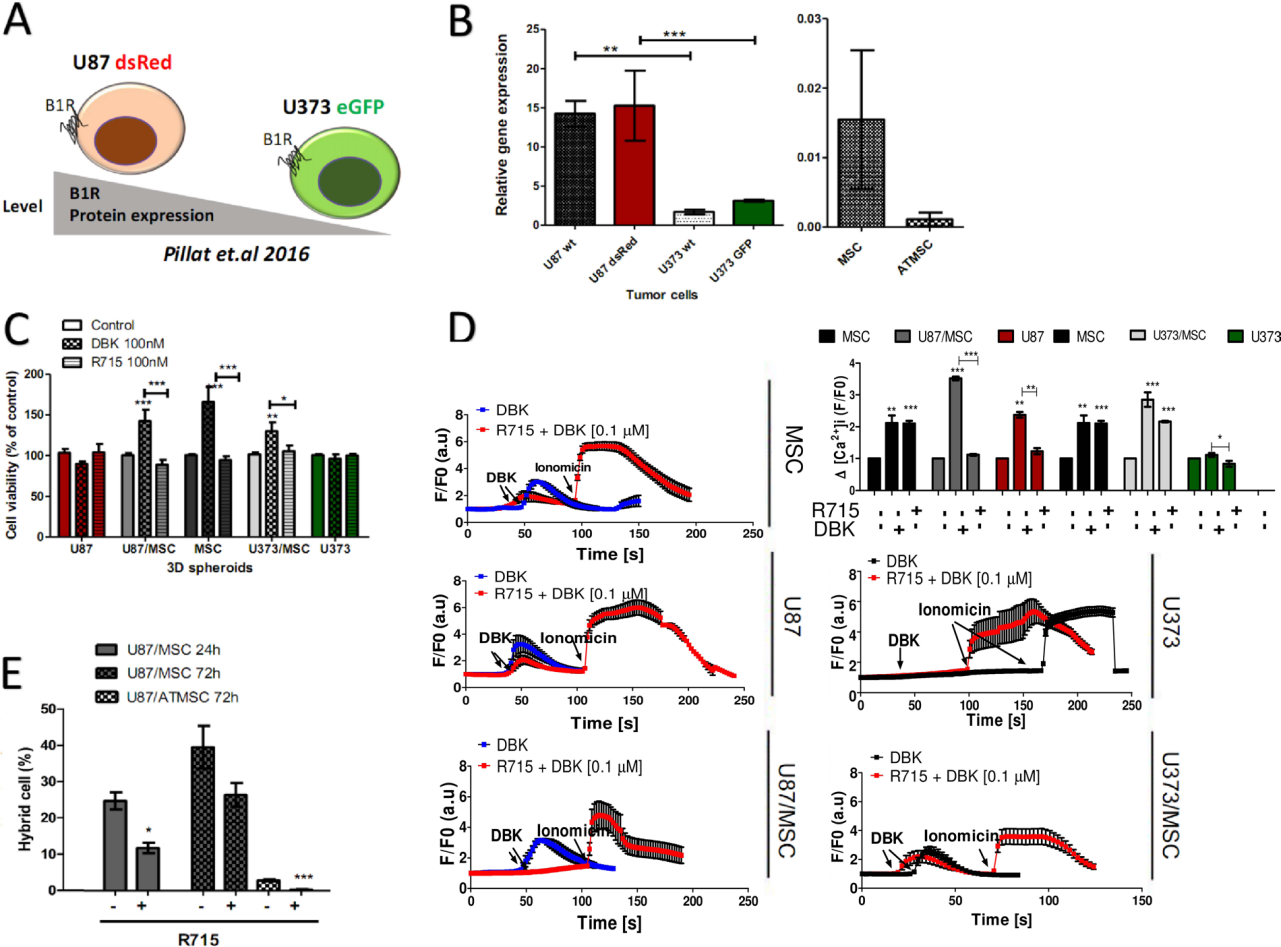
Fusion of GBM cells with BM-MSC and AT-MSC cells is associated with kinin-B1 receptor (B1R) expression and activity. (**A**) Scheme for B1R protein expression in GBM cell lines based on our previous report by Pillat *et al*.^21^. (**B**) Quantification of relative B1R mRNA expression levels in GBM cells (as indicated) and BM-MSCs (=MSC) and AT-MSCs as 2D monolayer mono-cultures, using qRT-PCR. (**C**) Quantification of cell viability of U87 and U373 cells and BM-MSC (MSC) in mono-spheroids and mixed spheroids for treatment with 100 nM des-Arg^9^-bradykinin (DBK; B1R agonist) and 100 nM R715 (B1R antagonist) for 5 days, as determined using the MTS assay with normalization to control conditions. (**D**) Intracellular calcium elevations [Ca^2+^]_i_ in MSC and glioma cells (U87-MG and U373 MG) in 2D mono and co-culture, mediated upon B1 receptors activation and inhibition with (R715). Prior to calcium imaging, cells were loaded with the Ca^2+^-sensitive fluorophore indicator Fluo-4AM. Non-stimulated cells were imaged for obtaining basal fluorescence values, followed by addition of 100 nM final concentration of des-Arg9-bradykinin (DBK) or R715. Kinetics of kinin receptor-induced [Ca^2+^]_i_ transients is shown in average (six trials) of the change in fluorescence (∆F/F) of DBK and R715 during time detection in the graph (right panel). The shown data are average responses of 10 cells. (**E**) Quantification of cells’ hybrid formation of U87dsRed cells with BM-MSC (MSC) and AT-MSC in 2D monolayer co-cultures for treatment with 100 nM R715 (B1R antagonist), after 1 and 3 days, measured by flow cytometry. Data are means ± standard deviation. *P < 0.05, **P < 0.001, ***P < 0.0001.

Induced expression of B1R in inflammation and cancers is well established^22^. Moreover, in our previous report^21^ we showed that that indirect and direct effects of BM-MSC on U87dsRed GBM cells were associated with increased expression of both B1R and B2R, which possibly triggers increased cell invasiveness. However, to date, no links between B1R and cell fusion or vesicle transfer have been reported. In view of this, the effects on cell-cell interaction were investigated here in terms of B1R expression and its agonist DBK and antagonist AcLys[D beta Nal^7^, Ile^8^]desArg^9^-bradykinin (R715), in both U87dsRed and U373eGFP GBM cells in 2D co-cultures with BM-MSC and AT-MSC. Previously, we have also showed higher B1R expression in U87 than U373 cells, and their labelled derivatives^21^, as shown in Fig. 5A (left). Contrary to these GBM cells, both BM-MSC and AT-MSC showed lower B1R expression, although higher B1R expression was seen for BM-MSC *versus* AT-MSC (Fig. 5B, right). The B1R agonist (DBK) and antagonist (R-715) were used at the effective dose of 100 nM for B1R activation and chronic inhibition, respectively^31^. As also shown previously, these are not cytotoxic for any cell types and under any condition tested (Fig. 5C). Moreover, DBK enhanced the viability/metabolic activity (measured using spheroids and the MTS assay), and thus promoted the proliferation of MSC mono-spheroids and both U87/BM-MSC and U373/BM-MSC as mixed spheroids. Figure 5D shows that U87/BM-MSC and U373/BM-MSC 2D co-cultures responded to B1R agonist stimulation with increased free cytosolic calcium ion concentrations, as compared to the 2D mono-cultures, and that this was effectively blocked by the B1R antagonist. Figure 5E addresses the association between B1R activation and cell-cell interaction events in 2D mixed co-cultures using the R715 antagonist of B1R, decreasing the number of cell-cell interaction that reduced heterotypic U87dsRed/AT-MSCeGFP hybrids formation from 3.0 ± 0.4% to 0.35 ± 0.21%, while for U87dsRed/BM-MSCDiO in 2D co-cultures the reduction was from 41.4 ± 15.1% to 27.6 ± 5.9% after 72 h (Fig. 5E and Supplementary Fig. 2). This also occurred for U87dsRed/AT-MSCeGFP 2D co-cultures, although these events were relatively rare compared to BM-MSCDiO. Interestingly, higher expression of B1R in U87 cells than in U373 cells also correlated with lower frequency of cell-cell interaction events in U373 cells in 2D co-cultures with BM-MSC (Fig. 3C). Taken together, negative modulation of B1R activity resulted in reduced heterotypic fusion and vesicle transfer between GBM cells and MSC in these 2D co-cultures. Moreover, we demonstrated that the frequency of cell-cell interaction events and expression of B1R differ in GBM cells of different subtype, being lower in U87 cells with a more proneural transcriptomic type than in U373cells, expressing typical mesenchymal transcriptome. Cell-cell interaction also depends on the tissue origin of MSC.

### Invasion of U87 glioblastoma cells in co-cultures with BM-MSC is affected by B1R activity

**Figure 6:**
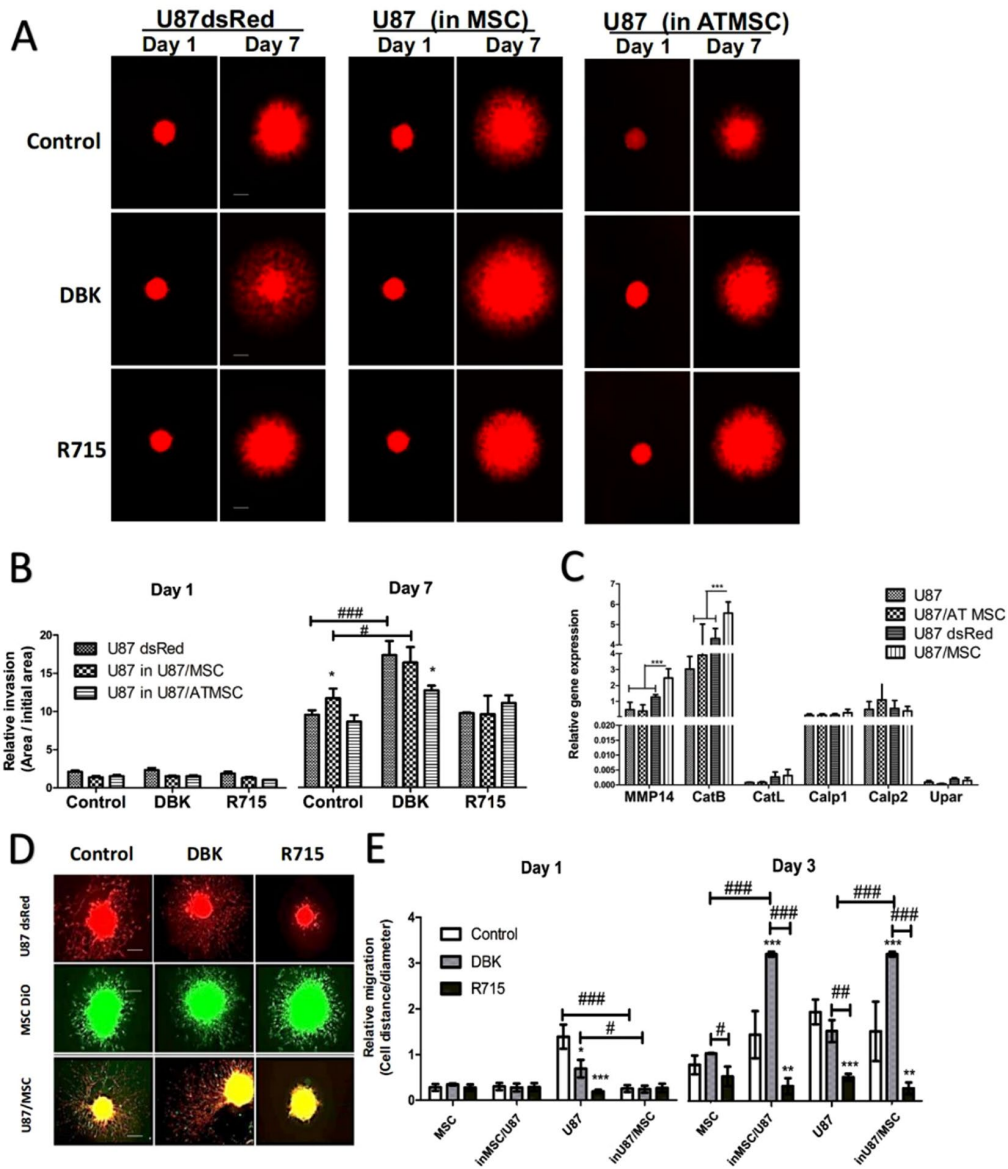
Bone-marrow (BM) MSC and adipose tissue (AT) MSC differentially affect U87 cell invasion responses to bradykinin receptor 1 (B1R) activation. (**A**) Representative images of U87dsRed cell growth and invasion with BM-MSC (=MSC) and AT-MSC as mono-spheroids and mixed spheroids for treatment with 100 nM DBK (B1R agonist) and 100 nM R715 (B1R antagonist), after 1 and 7 days. The spheroids were embedded in Matrigel (see Methods). Scale bar, 250 µm. (**B**) Quantification of the relative invasion as derived from data illustrated in (**A**), as the ratio of the invasion area *vs* initial spheroid area, after 1 and 7 days. (**C**) Quantification of relative gene expression (mRNA levels) of selected invasion-related proteases in U87 cells and BM-MSC (=MSC) and AT-MSC in 2D monolayer cultures and co-cultures, after 1 and 7 days. (**D**) Representative images of U87dsRed cells (red) and BM-MSC (MSC; green) migration/invasion from mono-spheroids and mixed spheroids, for treatment with 100 nM DBK (B1R agonist) and 100 nMR715 (B1R antagonist), after 3 days. The spheroids were embedded in Matrigel in 24-well plates. Scale bar, 250 µm. (**E**) Quantification of relative migration as derived from data illustrated in (**D**), calculated as the ratio of cell distance vs spheroid diameter for each time interval (see Methods). Data are means ± standard deviation (n = 3). *P < 0.05, **P < 0.001, ***P < 0.0001.

The question was whether differential B1R expression in U87dsRed cells, BM-MSC and AT-MSC (Fig. 5B) would also mirror their differential agonist (DBK) and antagonist (R715) invasion responses. When mono-spheroids and mixed spheroids of U87dsRed cells and both BM-MSC and AT-MSC were incubated for 7 days they showed enhanced invasiveness of U87dsRed cells out of the spheroids, and this was further stimulated by DBK in coculture with BM-MSC (Fig. 6A,B). Treatment with the antagonist R715 efficiently abrogated this effect in U87dsRed mono-spheroids and U87/BM-MSC mixed spheroids, whereas no such effect was observed in U87/AT-MSC mixed spheroids. Accordingly, in U87/BM-MSC 2D co-cultures, gene expression analysis revealed elevated levels of the pro-invasive proteolytic enzymes matrix metalloprotease (MMP)14 and cathepsin B, but not of other proteases, such as cathepsin L, calpains 1/2 and the urokinase receptor when comparable with coculture U87/AT-MSC (Fig. 6C). Furthermore, cell migration of U87dsRed cells and BM-MSC from mono-spheroids and mixed spheroids was quantified after 3 days of treatment with DBK and R715 (Fig. 6D,E) DBK significantly increased the migration of U87dsRed cells and MSC, whereas the B1R antagonist R715 was a potent inhibitor of cell migration (Fig. 6E). Taken together, these data suggest that B1R activation may play a role in the regulation of cell-cell interactions between GBM cells, which express B1R receptor, and MSC. B1R/DBK-mediated activation was correlating with enhanced migration/invasion rates of GBM cells and MSC out of co-cultures. This is particularly relevant in co-cultures with BM-MSC, which affected U87 transition (transdifferentiation) to a more mesenchymal phenotype markers (Fig. 4D,E).

## Discussion

Glioblastoma are malignant brain tumours that have the worst prognosis and high recurrence rates because of the tumour aggressiveness and resistance to chemotherapy and radiotherapy. In the tumour microenvironment, the interactions between tumour and stromal cells have been shown to modulate GBM aggressiveness and resistance to therapy^1^. The presence of non-neoplastic cells within the tumour stroma and studies that have traced the origins of such cells in animal models suggest that these cells participate also in tumour formation^6^. These cells provide the so-called inter-tumour heterogeneity, or tumour non-autonomous heterogeneity, and may significantly affect the tumour cell environment^32^, thus becoming an important target in cancer therapies. On the other hand, it is known that glioma stem cells can give rise to autonomous cellular tumour heterogeneity within different GBMs that have the same clinical appearance. Verhaak *et al*.^33^, and later several other studies^6,34^, postulated four different GBM transcriptome subtypes that appeared to co-exist within any single tumour. This has been recently demonstrated by Rickylefs *et al*.^35^, showing that this heterogeneity suggests more aggressive tumours with worse prognosis. Heterogeneous populations of tumour cells are exposed to heterogeneous populations of stromal cells, which might thus affect the tumour cells in very different ways. For studying this diversity, we used MSC as our model, which can infiltrate into the brain, presumably, but not necessarily, from the bone marrow. The tropism to neoplasia of these cells has been well documented^36^, which was also the basis for preclinical studies of MSC as vectors for cell-based cancer therapies^7,8^. Thus, their effects on heterogeneous populations of tumour cells should be considered before the application of any MSC therapies.

Recently, we demonstrated GBM cell-type-specific responses to BM-MSC in 3D direct co-cultures, where BM-MSC inhibited invasion of U87 cells, while enhancing invasion of U373 cells after 4 days^11^. We assumed that this was due to significant differences in the intrinsic gene expression profiles of these two GBM cell lines, which show less (U87) and more (U373) mesenchymal-like features. Here, we also found differential proliferation responses of these U87 and U373 cell lines in mixed spheroids with BM-MSC, as evaluated also by the cell *versus* spheroid size ratios. Although both U87 and U373 cells showed increased proliferation rates upon this direct contact with BM-MSC, the predominance of U87 cells, and to a lesser extent of U373 cells, over BM-MSC in these mixed spheroids was noted over 5 days, in parallel with reduced proliferation and size of the BM-MSC.

These data can be explained by the observed high rate of cell-cell interaction events between these GBM cells and BM-MSC, with levels of 25% and 3% for the U87 and U373 cells, respectively. Cell fusion has a crucial role in pathophysiological processes such as cancer, where it represents an efficient means of rapid phenotypic evolution, through the production of cells with new properties at rates that exceed random mutagenesis^36–38^. Consistent with our findings, higher fusion rates have also been observed in exponentially proliferating cells, compared to slowly proliferating cells^39,40^. These previous studies also indicated that cell hybrids formed by spontaneous fusion between GBM cells and normal hamster cells *in vivo* and showed modified GBM traits. Mercapide *et al*.^10^ also showed that homotypic U87 and heterotypic U87/fibroblast hybrids had higher proliferative activity, which resulted in a larger fraction of polyploidy in tumour cells, which were shown to arise from cell fusion. This is similar to what we observed in the U87/BM-MSC 2D co-cultures, which were associated with increased heterotypic fusion rate and increased size of the U87/BM-MSC, which also contained higher numbers of polyploidy cells compared to the U373/BM-MSC. BM-MSC might have an impact on giant cell formation, as we also noted increased numbers of giant U87 cells. We speculate that these might arise by homotypic fusion among the U87 cells when exposed to the BM-MSC, as we have also already reported on giant U87 cells when these were exposed to BM-MSC conditioned-medium alone^9^. The heterotypic hybrids might be transformed into viable re-programmed cells, and are emerging as a plausible source of increased tumour heterogeneity *in vivo*^41^, a process that is also stimulated by chemotherapy^42^. Such cells might contribute to cancer progression and metastasis, due to the genetic instability caused by their polyploidy^43,44^. Altogether, our data imply that even in the same tumour, cells of different tumour subtypes are differentially affected and more or less fuse with infiltrating BM-MSC, what accounts for their differential proliferation and polyploidy, mimicking the observations for GBM *in vivo*^32^.

Tumour cells release different types of vesicles including extracellular microvesicles (MVs) and microparticles, membrane blebs, apoptotic bodies, etc^45^. Similarly, nanovesicles, transferring proteins, lipids and various types of RNAs to neighbouring cells have been described for MSC in regenerative medicine^7,46^. MSC also release a number of growth factors, such as TGF-β, which may trigger alterations in transcription factors required to initiate process, similar to EMT^47,48^ in the neighbouring GBM cells resulting in enhanced migration, for example here out of the BM-MSC co-cultures, where we found increased levels of adherence junctions in U373/GBM cells^3^. Here we observed initial increase in the CD29^49^, which is an integrin β1 that is involved in EMT, in U87 GBM cells, but CD29 expression decreased after prolonged co-culturing from of 59% to 24% of, possibly being related to slightly enhanced invasion (Fig. 6). Heterotypic cell fusion was imaged here in the coculture, presumably as a result of the “invasion” of the MSC cells into the U87 cells (Supplementary Fig. 1). However, in related work by Breznik *et al*.^11^ we observed enhanced invasion of MSC, but not of U87 out of in U87/BM-MSC spheroids.

Here we also occasionally observed ‘cell in cell’ cytological features, as often observed in cancers, which is termed entosis^50^. Recently, Bartosch *et al*.^51^ demonstrated a similar phenomenon, termed cell cannibalism, where BM-MSC were shown to enter breast cancer cells in 3D co-cultures. This enhanced the survival of the cancer cells, but at the same time suppressed their tumorigenicity, and promoted their dormancy. As suggested by these authors, the MSC were mobilised from the bone marrow and migrated into the tumour environment, promoting the formation of tumour cell spheroids that were finally internalised by the tumour cells. Moreover, our data demonstrate a significantly higher cell-cell interaction of these U87 cells with the BM-MSC (about 40% of cells) *versus* the fusion rate with AT-MSC (only 3% of cells), which provides the first evidence that the tendency of cancer cell interaction with MSC depends on the MSC tissue origin. Such data are supported by the observation of Akimoto *et al*.^52^, and Del Fattore *et al*.^27^, who showed differential effects of BM-MSC, AT-MSC and umbilical cord blood MSC on glioma cells *in vitro*, but most likely, this reflects the *in-vivo* situation, as BM-MSC tropism to the brain tumours have been demonstrated in mice and humans^53^.

It is important to emphasize that the elevated number of double-stained cells in our experiments could also result from an exchange of microvesicles as well as transdifferentiation through. Recently, Vignais *et al*.^54^ pointed at the potential of MSC in modelling nanotubules for syncytium formation by rearrangement of the cytoskeleton and cell-to-cell transfer of mitochondria under physiological and pathological conditions, changing cell metabolism and functions related to cellular energy. The importance of cell fusions in tissue remodelling has been documented as a physiological process during regeneration^55,56^, to which MSC actively contribute.

Cell fusion is also triggered by chronic inflammation^55^, and 10-fold to 100-fold increases in cell fusion frequency have been reported in liver, brain and intestinal tissue under chronic inflammatory conditions^57^. Furthermore, cell integration through cannibalism of MSC by tumour cells would augment the liberation of pro-inflammatory factors^58^. Along with a plethora of other cytokines, this would induce expression of B1R^14,17,21,59,60^. As these processes are orchestrated by GBM-derived factors, which include cytokines, we explored the possibility of a link between B1R in GBM cell fusion and invasion under chronic inflammatory conditions. Inflammatory signals *per se* and increased cell fusion were shown to enhance tumour progression, as many cancer hybrid cells showed more metastatic behaviour^60^. Shichor *et al*.^3^ proposed that upon cell contact, molecular signals from MSC internalised by U373 GBM cells can result in transcriptional activation downstream of the mitogen-activated protein kinase/ERK cascade, which is known to regulate both cell motility and proliferation. We have demonstrated that the U87 cell invasion is suppressed by BM-MSC can be triggered by the addition of a B1R agonist (DBK), which is consistent with the very high B1R expression in these U87 cells. However, the induced invasiveness of U87cells in mixed spheroids with MSC appeared to be delayed, as this was observed between 1–4 but after long time of analysis with 7 days the invasiveness is observed in these co-cultures. This delay roughly coincides with the cell-cell interaction rate increase that was observed over this period of time. Consistent with the negligible B1R expression in U373 cells, no changes in cell invasion were noted upon either agonist or antagonist treatment of U373/BM-MSC spheroids (data not shown). We demonstrated that the fusion is diminished by B1R blocking/or low expression and are involved with invasiveness potential *via* its agonist DBK, to enhance this signalling via the PI3K/Akt and ERK1/2 pathways^61^. The functionality of B1R was shown here by stimulation of the intracellular calcium ion flux. Our previous data showed that treatment of U87 GBM cells with bradykinin resulted in down-regulation of B1R and B2R expression in BM-MSC, with simultaneous expression up-regulation of B1R and B2R in U87 cells. This suggests that bradykinin mediates the information flow between these cells, to some extent regulating tumour progression and invasion^21^. As we did not detect significant changes in the invasiveness-related proteases in U87 cells, we believe that the MSC- and DBK-induced migration is related to the changes in intracellular calcium transients, through a mechanism described by Seifert and Sontheimer^62^.The intracellular calcium ion transients in GBM cells *in vitro* induced the formation of small bleb-like protrusions at the plasma membrane, which stimulated an amoeboid phenotype of cell migration that was unrelated to proteolytic activity and ECM degradation^63^.

In conclusion, this study has shown that the frequency of cell-cell interaction by heterotypic fusogenic events, vesicle transfer and entosis in the two established GBM model cell lines, U87 and U373 cells, preferentially with BM-MSC (vs. AT-MSC) is related to cell genotypes /phenotypes, which include differential B1R expression levels. The mechanisms by which these cells respond to cues from the micro-environmental, such as infiltrating MSC, with induce epithelial to mesenchymal transition and activate invasion-related proteases in less-invasive GBM cells, such as these U373 cells, which are not higher cell-cell interaction. In contrast, the more invasive and fusogenic U87 cells need B1R activation, which would most likely depend on a more or less inflammatory environment within a tumour *in vivo*. Altogether, our study supports the on-going exploration of B1R agonists and antagonists as target candidates in the development of adjuvant approaches to GBM therapy. Secondly, our study indicates the need to further and carefully explore MSC as potential candidates for cell therapies, particularly with respect to tumour cell autonomous heterogeneity.

## Materials and Methods

### Cell lines and culture maintenance

The U87 and U373 human GBM cells lines were obtained from American Type Culture Collection (USA) and regularly authenticated, according to Torsvik *et al*.^64^. GFP-transfected U373 cells (U373eGFP) and dsRed-transfected U87 cells (U87dsRed) were established as previously stated^3,21^. Cells were grown in complete growth medium that consisted of Dulbecco’s modified Eagle’s medium (DMEM) supplemented with 10% foetal bovine serum (FBS), non-essential amino acids, 4mML-glutamine, 100 U/mL penicillin and 100 μg/mL streptomycin (Gibco). The U373eGFP and U87dsRed cells were routinely propagated in the medium with additional 1mg/mL G-418 (Sigma-Aldrich). The cells were cultured at 37 °C, in 5% CO_2_, and the medium was changed every 3 days. These cells were passaged after reaching 75% confluence, and plated for culture maintenance at a density of 15000 cells/cm^2^.

Human bone-marrow MSC derived from bone marrow of a healthy volunteer (MSC-2) were purchased from Lonza Bioscience Walkersville (Walkersville, MD, USA). The AT-MSC transfected with GFP were generously provided by Prof. Massimo Dominici (University Hospital of Modena, Italy). After resuscitation, the cells were plated and grown in the same medium as the GBM cells, however, without G-418. The MSC were passaged after reaching 75% confluence at a density of 7000 cells/cm^2^. BM-MSC of passages 8–12, and U87/U87-dsRed and U373/U373-GFP GBM cells of passages 40–58 were used, in culture medium containing 10% FBS.

### Two-dimensional and three-dimensional cell co-cultures

For 2D direct cell co-cultures (i.e., cell types in direct contact with each other), GBM cells and MSC were plated into 6-well plates at a 1:1 ratio, and cultured for 1–7days. Direct co-cultures of U87/BM-MSC, U87dsRed/BM-MSC, U373/BM-MSC, U373eGFP/BM-MSC and U87-dsRed/AT-MSC-GFP were prepared using different plates and time intervals, and also always at a 1:1 ratio. For preparation of the spheroid cultures (mono-spheroids, mixed spheroids), the corresponding cells (as 1:1 cell mixtures) were seeded in 200 µL complete DMEM growth medium containing 4% methylcellulose, into 96-well U-bottomed plates (2.5 × 10^3^cells/well; Corning, Life Sciences, MA, USA). They were centrifuged for 90 min at 850 × *g* and left in culture for 1 day at 37 °C under 5% CO_2_, to form one spheroid in each well. In the mixed spheroid or “hybrid cell, GBM and MSC cells can fuse, what we defined as “heterotypic fusion”, or “hybrid cells” to distinguish these form “homotypic fusion”, which occurs often between cancer cells, resulting in aneuploidy (polyploidy). Fusogenicity is the ability of cells to fuse the membranes and form binuclear cells (heterokaryon) or a giant nuclei, resulting from nuclear fusion (synkaryon)^10,28^. The fusogenicity is quantified by the % of fusion events observed in defined cell population.

### Proliferation analysis

About 20–24 mono-spheroids or mixed spheroids were transferred into 2 mL microcentrifuge tubes (Eppendorf) after 1, 3 and 5 days of culture, dissociated with trypsin/EDTA (Corning, Life Sciences, USA), blocked with growth medium (DMEM plus 10% FBS), centrifuged, and resuspended in 100 µL phosphate-buffered saline for cell counting. The cell numbers, diameters and volumes were determined using a handheld automated cell counter (Scepter; Merck-Millipore, Billerica, MA, USA), using 40 µm and 60 µm cell filters for the sensor. The number of cells per spheroid in each tube was determined by dividing the total number of cells counted in each tube by the initial number of spheroids. For proliferation determination of the different cell types in coculture (U87dsRed, U373eGFP and BM-MSC), a parallel analysis was performed by flow cytometry with the same samples. As different cell types were distinguished by flow cytometry, it was also possible to analyse proportions of each cell type in co-culture and spheroids. The data were gathered from three independent experiments. The significance of differences between mono-culture and co-culture conditions was determined using ANOVA two-way and Student’s t-tests, and P < 0.05 was considered as significant.

### Determination of cell doubling time

Following the use of the automated cell counter (Scepter; Millipore), the doubling time of cell proliferation at each assayed time point was determined using Equation (1) (Roth V. 2006 Doubling Time Computing, Available from: http://www.doubling-time.com/compute.php):

1 $${\rm{Doubling\; Time}}={\rm{Duration}}\,\times \,\mathrm{log}(2)/\mathrm{log}({\rm{Final}}\,{\rm{Concentration}})-\,\mathrm{log}({\rm{Initial}}\,{\rm{Concentration}}).$$

This equation was used for the calculation of the doubling time of each cell population.

### Cell cycle and DNA content analyses

Bone-marrow MSC were pre-loaded with DiO and DiL, and were cultured in 2D co-cultures together with U87dsRed or U373eGFP cells, for 24 h or 72 h. The cells were then harvested, and washed with DMEM without serum, and 5 × 10^5^ cells/mL were incubated with 5 µM Vibranty Dye Ruby (Life Technologies) for 15 min, according to the instructions of the supplier. Cell cycle analyses were performed with a flow cytometer (FACS Calibur; Becton and Dickinson, CA, USA) using 488 nm and 633/5 nm as excitation wavelength, and >670 nm as emission wavelength (linear scale). Fluorescence emission data of DiO or GFP (FL1,488 nm excitation, 533/30 nm emission band-pass filter) and fluorescence emission data of dsRed or DiL (FL2, FL1, 488 nm excitation, 585/40 emission band-pass filter) were used to determine the gates for the different cell populations. Three independent experiments were performed. The data were analysed using the FlowJo V10 software (FlowJo, LLC, OR, USA) to determine the fractions of cells in the sub-G0/G1, G0/G1, S and G2/M phases of the cell cycle.

### Spheroid viability measurements (MTS assay)

Cell viability within the spheroids was determined using the MTS assay. After 1, 3 and 5 days, for all control and treated spheroids grown in U-bottomed plates with 100 uL media/well, 20 μLMTS agent was added (Promega, Fitchburg, WI, USA). The spheroids were incubated at 37 °C for 3 h, and their absorbance was measured at 490/630 nm using a microplate reader (Genios; Tecan, Bradenton, FL, USA). The measurements were performed in triplicate and repeated in three independent experiments.

### Flow cytometry analysis of 2D coculture and mesenchymal-specific markers

Different types of mixed spheroids (U87dsRed/BM-MSC, U373eGFP/BM-MSC) were cultured for 1, 3 and 5 days. They were then dissociated, and single-cell suspensions were subjected to flow cytometry analysis (FACS Calibur; BD Biosciences, NJ, USA). Forward and side light scatter signals were used to detect the size of each cell population in the co-cultures. GBM cells and MSC were discriminated based on the GFP (FL1-H) or dsRed (FL2-H) fluorescence signals, following compensation for eliminating non-specific signals.

Experiments for quantification of MSC-specific markers, CD29, CD90 and CD105, were performed according to Pillat *et al*.^21^ MSC were pre-loaded with DiO and then seeded in monocultures or 2D co-cultures together with U87dsRed cells for 72 h. Cells were dissociated from the flasks using trypsin and FBS. Single cells were fixed for at least 20 min in 4% para-formaldehyde, washed with PBS 1% FBS, and incubated with antibodies anti-CD90 APC (5 µL:100 µL; BDPharmingen; cat 559862; FL4) and anti-CD29 PE-Cy^TM^5 (20 µL:100 µL; BD Pharmingen; cat 559882; FL3) or anti-CD105 APC (5 µL:100 µL; BD Pharmingen; cat 562408; FL4) for 30 min at room temperature. Cells were washed with PBS and, then analysed in a flow cytometer. A minimum of 10,000 events was acquired per sample, and the data were displayed at logarithmic scales and analysed with the FlowJo V10 software.

### DsRed positive cell selection

MSC were pre-loaded with DiO, and were seeded in monocultures or 2D co-cultures together with U87-dsRed cells for 8 days, with the medium change every 2 days. Control monoculture of U87dsRed and BM-MSC were set as well. On the 6^th^ day, the 3^rd^media change was performed with the media supplemented with geneticin (G418) at 2 mg/ml to select for U87dsRed cells only as only those cells contain the Neo resistance cassette in their genome. On the 8^th^ day the surviving cells out of U87dsRed monoculture and U87/BM-MSC coculture were collected and fixed with 4% paraformaldehyde for flow cytometry analysis (no BM-MSCs grown in monoculture survived the gentamicin treatment). Fixed cells were immunostained for CD29, CD90 and CD105 mesenchymal markers and analysed by flow cytometry.

### Three-dimensional invasion assay

The 3D invasion assay was performed as described previously^65^. Briefly, the spheroids were covered with 5 mg/mL Matrigel (BD Bioscience) into 96-well U-bottomed plates. After a 45-min incubation at 37 °C and under 5% CO_2_, the embedded spheroids were covered with DMEM with 10% FBS (control spheroids) and 100 nM DBK or R715 were added (treated spheroids). The initial distance of invasion (T0) was measured following an overnight incubation, and then on each successive day, for a total of 7 days. The distance was measured from the edge of the spheroid by using the NIS elements software 2.3 v (Nikon Instruments, Melville, NY, USA) and normalized to the size of the spheroid.

### Three-dimensional migration assay

The migration assay was performed as described previously^11^. Briefly, the spheroids were transferred to 24-well plates (TPP, Trasadingen, Switzerland) and covered with 5 mg/mL Matrigel (BD Bioscience, USA). After a 45-min incubation at 37 °C under 5% CO_2_, the embedded spheroids were covered with DMEM with 10% FBS (control spheroids) or 100 nM DBK or R715 were added (treated spheroids). Cell migration was determined by evaluating the distance between the cells and the edge of the spheroid over 3 successive days. Images were analysed with the NIS elements software 2.3 v (Nikon Instruments, Melville, NY, USA). Migration distances were normalized to the spheroid diameter.

### Fusion, vesicle transfer and entosis analysis

Bone-marrow MSC were pre-stained for 30 min at 5 µM DiO or DiL (final concentrations; Thermo Fisher, MA, USA) at 37 °C under 5% CO_2_ in a humidified incubator. They were then washed twice with 1 mL phosphate-buffered saline and set-up for a 2D co-cultures with U87-dsRed or U373-GFP cells in 6-well plates, for 24 h and 72 h, as control cells and those treated with 100 nM R715 (antagonist). AT-MSC-GFP were used directly in co-cultures with U87dsRed cells under control and treatment conditions for 72 h. After these incubations, the cells were dissociated with 1 mL 0.25% trypsin/EGTA (Gibco-Thermofisher), blocked, and resuspended in phosphate-buffered saline for flow cytometry analysis (FACS Calibur; BD Biosciences, USA). Ten thousand events were acquired per sample, and the data were displayed on logarithmic scales. Forward and side light scatter signals were used to exclude dead cells and debris. DiO/GFP- and DsRed/DiL-positive cells were determined as the percentages of cells stained/with positive expression using the FL1-H and FL2-H channels, out of the total cells in each co-culture sample (U87dsRed/MSC-DiO, U373eGFP/MSC-DiL). Mono-culture control cells were used to determine the signal background and to set the gate boundaries. The data were analysed using the FlowJo V10 software (FlowJo, LLC, OR, USA).

Hyperploids cell populations were analysed and quantified using Vybrant Dye Cycle with the FL-4 (640 nm excitation, 675 nm emission, 25 nm band pass filter;675/25 nm). The diploid (2n) and <4n (diploid continuous = tetraploid, polyploid, aneuploid cells) populations were calculated based on (*A*) area × width (*W*). These parameters (*A* *vs*. *W*) were primarily set to discriminate single cells passing through the flow cell from cells that might not have spatial separation when passing through the flow cell of the cytometry; i.e. doublets (aggregated cells). Doublets will give an increased width measurement (26% increase for every doubling of volume of a cell, based on a spherical arithmetic model). Diploid cells result in increased area fluorescence signals without any increase in width fluorescence signals.

### Cell treatment

The agonist Des-Arg^9^-bradykinin (DBK) and the antagonist AcLys[D beta Nal^7^, Ile^8^]desArg^9^-bradykinin(R-715) (both from Tocris, Fisher Scientific, Pittsburg, PA, USA) were added to the growth medium or matrigel of the 3D spheroids at final concentrations of 1 to 1000 nM. The spheroids were incubated with these for 24 h up to 168 h (7 days).

### Viability MTT assay in BR1 activity testing

The effects of the B1R DBK agonist and R715 antagonist on the survival of GBM cells (U87dsRed, U373eGFP) and MSC in 2D monolayer cultures were determined using MTT (Sigma-Aldrich, St. Louis, MI, USA). The experiments were performed in 96-well plates (TPP; 8 × 10^3^ cells/well). Control and treated cells were incubated with MTT at a final concentration of 1 mg/mL for 3 h. Then the cells were centrifuged, the medium was removed, and 50 µL isopropanol was added, with agitation for 1 min. The optical density of each sample was measured at 570/630 nm using a spectrophotometer microplate reader (Genios; Tecan). Three independent experiments were performed, with six replicate wells per condition. The data are given as percentages of viable treated cells, relative to untreated control cells.

### Kinin-B1 receptor gene expression by real-time qPCR

Total RNA was isolated from three biological replicates of 2D monolayer mono-cultured U87, U373, U87dsRed and U373eGFP GBM cells, as well as BM-MSC and AT-MSC, using the Trizol reagent, according to manufacturer instructions (Invitrogen, Paisley, UK). The mRNA integrity was confirmed (Agilent 2100 Bioanalyser; Agilent Technologies, Santa Clara, CA, USA). The cDNA was generated from 1.0 µg total RNA using the High-Capacity cDNA Reverse Transcription kits (Applied Biosystems, Hilden, Germany). The relative quantification of gene expression levels of B1R was carried out using real-time PCR (ABI 7900 HT Sequence Detection System; Applied Biosystems, ThermoFisher, Waltham, MA, USA). These real-time PCR reactions were performed using 1:10 dilutions of each cDNA (1 µg/well), added to TaqMan Universal PCR Master Mix and TaqMan Gene Expression Assays (all from Applied Biosystems): for B1R-Hs00664201_s1 *versus* the amplification of glyceraldehyde 3-phosphate dehydrogenase (Pre-Developed TaqMan Assay Reagent No. 4310884E) as internal control. Here, 1, 1:10, 1:100, 1:1000 and 1:10000 dilutions of the template sample were used for relative B1R mRNA quantity determination in the samples. The SDS v2.2 software was used (Applied Biosystems) to analyse the data obtained from the TaqMan Gene Expression Assays. Independent experiments were performed in duplicate and repeated three times. Statistical significance between different cell lines was determined by two-tailed Student’s t-tests, and P < 0.05 was considered significant.

### Calcium Imaging

U87 and U373 GBM cells and human MSCs were seeded into 4 wells µ-Slide chamber ™ Ibidi (Munich-Germany) for 72 h at a density of 16.8 cells/cm^2^. For direct co-cultures, the mix of MSC and U87-MG cells (1:1 ratio) were seeded and cultured for 72 h. Prior to calcium imaging, the cells were washed with PBS and loaded with 5 mM of Fluo 4-AM for 30 min at 37 °C in serum-free medium containing 0.06% of the non ionic surfactant pluronic acid F-127 (Sigma Aldrich), as published previously^21^. Cells were rinsed with serum-free medium and equilibrate in the chamber for 15 min before calcium images were recorded with Nikon fluorescence imaging microscope (ECLIPSE-TiS) (Nikon, Melville, NY) equipped with a 14 bit high-resolution CCD camera CoolSNAP HQ2 (Photometrics, Tucson, AZ) and analyzed with NIS-Element software 2.3 v(Nikon). Fluorescence excitation was done with a xenon lamp at wavelength 488 nm and the light emission was collected at 520 nm using a bandpass filter at 515–530 nm. Time kinetics of free intracellular calcium ([Ca^2+^]i) variations were constructed from over 300 images collected in 1-s intervals. The fluorescence intensities (F) were calibrated in a solution containing 5 mM ionophore (Fmax) and 10 mM EGTA (Fmin) to provide an estimation of the absolute change in the intracellular calcium fluorescence using the following equation ∆F/F_min_. The cells were stimulated by 100 nM des-Arg^9^-bradykinin (DBK) or 100 nM R715 following of DBK.The presented traces are average variations over time of at least 20 single cells.

### Determination of proteases gene expression by real-time qPCR

Total RNA was isolated from 2D monolayer mono-cultured U87 cells and mixed co-cultures (U87/BM-MSC, U87/AT-MSC) using the TRIzol reagent (Life Technologies). Following DNase I treatment, 1 µg RNA was reverse transcribed into cDNA using SuperScript II Reverse Transcriptase (Life Technologies). Quantitative SYBR Green real-time PCR was performed (StepOnePlus; Life Technologies). Each 25 µL SYBR Green reaction consisted of 25 ng cDNA, 12.5 µL 2 × SYBR Green Universal PCR Master Mix (Life Technologies), and 200 nM respective forward and reverse primers. Unless otherwise stated, the primer sequences (Table 1) were designed using Primer-BLAST designer (https://www.ncbi.nlm.nih.gov/tools/primer-blast/). Real-time PCR was performed using the temperature protocol of 50 °C for 2 min, 95 °C for 10 min, followed by 40 cycles of 95 °C for 15 s and 60 °C for 1 min. The data were analysed following a dissociation curve protocol for evaluation of the specificity of the amplicon produced in each reaction. A distinct peak indicated that a single DNA sequence was amplified during the PCR. Standard curves were determined for each primer set and cDNA sample, to determine the efficiency of the reaction. Independent experiments were performed in triplicates for each gene analysed, and repeated three times. Glyceraldehyde-3-phosphate dehydrogenase gene expression levels were used as the endogenous control for relative quantification of gene expression levels (Table 1).

**Table 1:** Primer sequences of the cDNAs coding for the proteases receptors and proteases-related enzymes used for the real-time PCR.

**Gene**			**Primers: forward (F)/reverse (R)**
**Access number**	**Species**	**Name**	
NM_004995.3	*Homo sapiens*	Matrix metallopeptidase 14 (MMP14)	F: 5′GCTCTCTTCTGGATGCCCAA3′ R: 5′ GTACTCGCTATCCACTGCCC 3′
NM_001908.4	*Homo sapiens*	Cathepsin B (CTSB)	F: 5′TGCAATGTCACAACCTCTCTGA3′ R: 5′ TAGGAGGCAATCTGCAAACCA3′
NM_001257973.1	*Homo sapiens*	Cathepsin L (CTSL), transcript variant 5	F: 5′CAAGTGGAAGGCTGCAATGG3′ R: 5′ AGTCCAGGCCTCCATTATCCT3′
NM_001198868.1	*Homo sapiens*	Calpain 1 (CAPN1)	F: 5′ CCATAGACATCTCCAGCGTTCT3′ R: 5′CACGTTGTTCCACTCTGAGGA 3′
NM_001748.4	*Homo sapiens*	Calpain 2	F: 5′ CTCAACCAGGACTACGAGGC3′ R: 5′ TGATAAACTGGGGGTCAGCG 3′
NM_001005377.2	*Homo sapiens*	Plasminogen activator, urokinase receptor	F: 5′ACTCACGGACCGAAAAACCA3′ R: 5′TCCAGGTCTGGGTGGTTACA3′
GAPDH	*Homo sapiens*	Glyceraldehyde 3-phosphate dehydrogenase	F: 5′CCTCCCGCTTCGCTCTCT3′ R: 5′GCTGGCGACGCAAAAGA3′

### Live cell photomicrography

Images of live cells in the monolayers and as spheroids under control conditions and after addition of the agonist or antagonist (100 nM) were recorded after 24, 48, 72, 96 and 168 h using an inverted microscope (Nikon) coupled to a digital camera. The micrograph images were processed using the Software NIS-Elements 2.3 v software, for measuring the areas and cell distances, and for merging the fluorescent signals (GFP, dsRed, DiO, DiL). Five micrograph images were taken per experimental condition.

In time-lapse imaging, the cells were pre-loaded with respective dye, as described in fusion analysis. Cells were plated into 6 well and co-cultured for 48 h. All images of living cells were assisted by the Software NIS-Elements 2.3 v software, for automated focus and loop time was set at 7 min for 5 h.

### Statistical analyses

Statistical comparisons between the different conditions were carried out using Student’s t-tests, one-way analysis of variance (ANOVA), or two-way analysis of variance with Bonferroni *post-hoc* tests, using the GraphPad Prism 5.1 software (Graph-Pad Software, La Jolla, CA, USA). Unless otherwise stated, all of the data are expressed as means ± standard error of the mean (SEM), from at least three independent experiments. For flow cytometry, a minimum of 10000 events was analysed per cell sample. The criteria for statistical significance were set at *P < 0.05 and **P < 0.001.

### Data availability statement

The data availability is in line with the Journal policy and Scientific Reports regulations and is available upon request.

## Electronic supplementary material

Supplementary information

Supplementary video 1

Supplementary video 2
