# Supplementary material for: Kinin-B1 Receptor Stimulation Promotes Invasion and is Involved in Cell-Cell Interaction of Co-Cultured Glioblastoma and Mesenchymal Stem Cells
Source: Sci Rep. 2018 Jan 22;8:1299. doi: 10.1038/s41598-018-19359-1 (PMC5777993; doi:10.1038/s41598-018-19359-1)
Supplement: Supplementary file 1 — Supplementary information [file 41598_2018_19359_MOESM1_ESM.doc]

**Kinin-B1 Receptor Stimulation Promotes Invasion and is Involved in Cell-Cell Interaction of Co-Cultured Glioblastoma and Mesenchymal Stem Cells**

***Mona N. Oliveira^1,2,3^,Micheli M. Pillat^1^, Helena Motaln^2^, Henning Ulrich^1,3^*and Tamara T. Lah^2,3,4^****

*^1^Department of Biochemistry, Institute of Chemistry, University of São Paulo, Av. Prof. Lineus Prestes 748, São Paulo - SP, 05508-000, Brazil*

*^2^Department of Genetic Toxicology and Cancer Biology, National Institute of Biology, Večna pot 111, 1000 Ljubljana, Slovenia;*

*^3^Jožef Stefan International Postgraduate School, Jamova ulica 39, 1000 Ljubljana, Slovenia*

*^4^Department of Biochemistry, Faculty of Chemistry and Chemical Engineering, University of Ljubljana, Večna pot 113, 1000 Ljubljana, Slovenia*


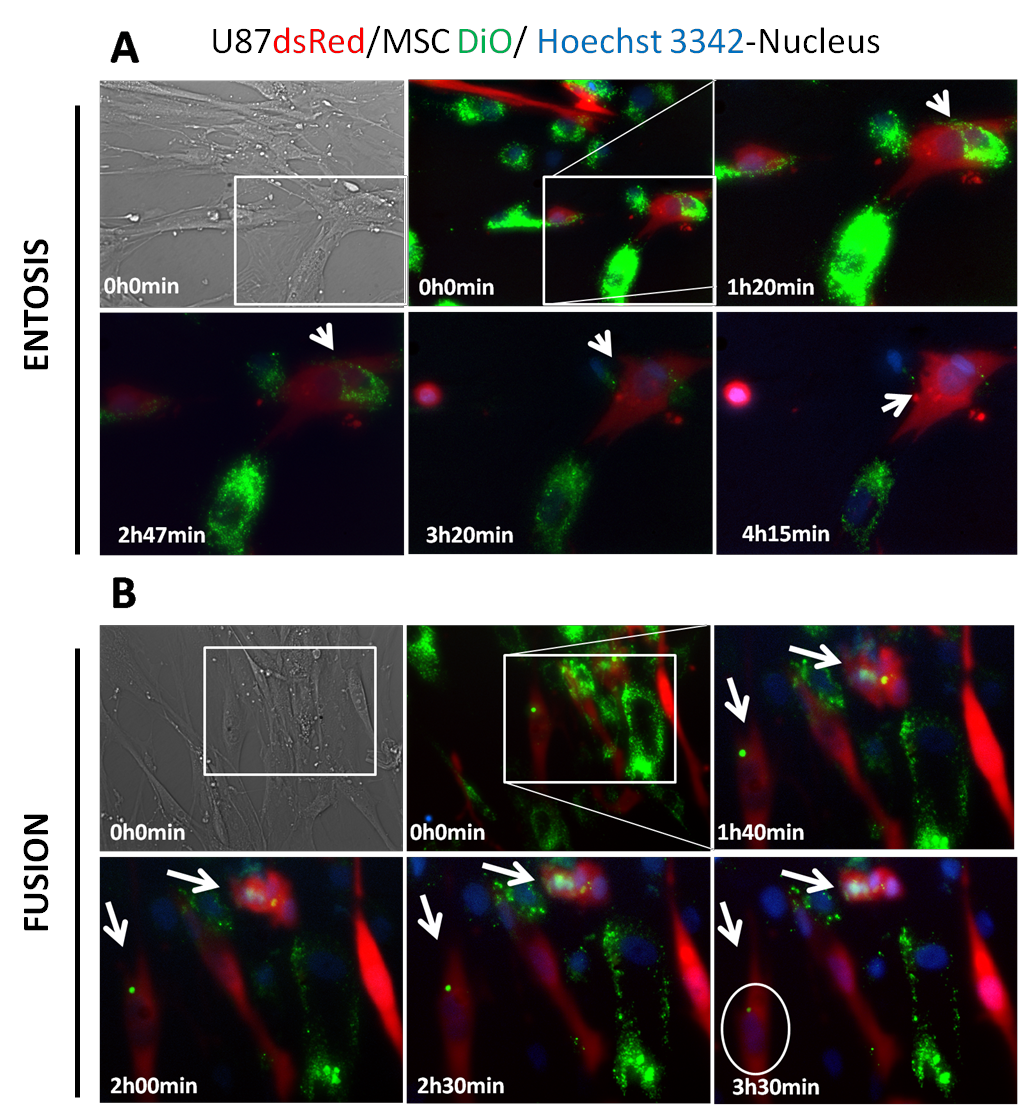


**Supplementary Figure S1. Time lapse imaging of U87dsRed/BM-MSCDiO co-culture shows entosis, cell fusion and vesicle transfer after 48h.** (A) Demonstration of the steps of process, similar to entosis of a BM-MSC DiO (green) and U87dsRed cells (red), following cells fusion event. (B)The time lapse demonstrate the steps of nuclear fusion. Fused cells progressively fuse the nuclei exhibiting a combined yellow staining marked by arrows. BM-MSC also secrete 6 large vesicle (in green) marked by a circle.


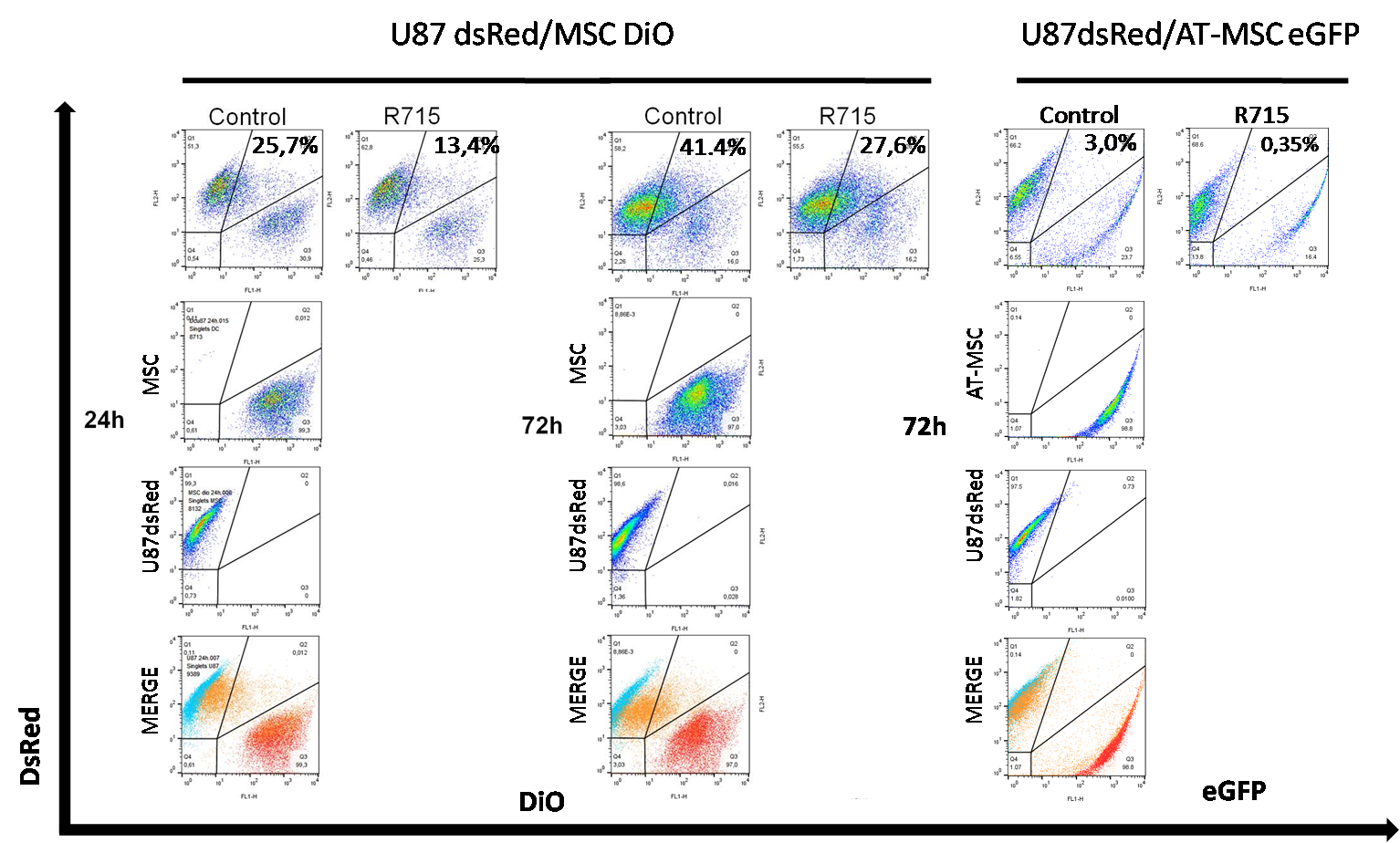


**Supplementary Figure S2. Dot-plots of U87dsRed/BM-MSCDiO and U87dsRed/AT-MSCeGFP 9 co-culture shows cell fusion and vesicle transfer after 24h and 72h treatment with R715**. Cell-cell interaction of U87 dsRed cell, BM-MSC DiO and AT-MSCeGFP were measured by flow cytometry. The representative dot-plots of control or treatment with R715 after 24h 12 and 72h are presented.
